# Pathogenic Variant in the 5’-Untranslated Region of *GCH1* and Clinical Heterogeneity in a Chinese Family with Dopa-Responsive Dystonia

**DOI:** 10.5334/tohm.974

**Published:** 2025-01-07

**Authors:** Yanting Li, Mingqiang Li, Lanqing Liu, Qiying Sun, Guang Yang

**Affiliations:** 1 Department of Geriatric Neurology, Xiangya Hospital, Central South University, Changsha, Hunan, 410008, China; 2 Department of Neurology, The First Affiliated Hospital of University of South China, Hengyang, Hunan, 421001, China; 3 National Clinical Research Center for Geriatric Disorders, Xiangya Hospital, Central South University, Changsha, Hunan, 410008, China; 4 Key Laboratory of Hunan Province in Neurodegenerative Disorders, Central South University, Changsha, Hunan, 410008, China; 5 Department of General Medicine, Xiangya Hospital, Central South University, Changsha, Hunan 410008, China

**Keywords:** GCH1 gene, Dopa-responsive dystonia, 5’-Untranslated region, phathogenic variant, phenotype-genotype correlation

## Abstract

**Background:**

Variants in the *GCH1* gene, encoding guanosine triphosphate cyclohydrolase, are associated with dopa-responsive dystonia (DRD) and are considered risk factors for parkinson’s disease.

**Methods:**

Comprehensive neurological assessments documented motor and non-motor symptoms in a Chinese family affected by DRD. Whole-exome sequencing (WES) was employed to identify potential mutations, with key variants confirmed by Sanger sequencing and analyzed for familial co-segregation.

**Results:**

The proband, a 50-year-old woman with a 10-year history of limb rigidity, abnormal posture, and a 23-year history of neck deviation, showed significant symptom improvement with levodopa treatment. Family evaluation revealed similar motor symptoms in four additional affected members, all responding well to levodopa. WES identified a *GCH1* variant (NM_000161.3: c.–22C > T) in the 5’-untranslated region (5’ UTR) in four symptomatic individuals (excluding deceased II-3). This variant likely affects translation by introducing an upstream initiation codon and open reading frame (uORF), leading to decreased BH4 levels and disrupted dopamine synthesis.

**Discussion:**

This study reports a pathogenic variant in the 5’ UTR of *GCH1* in a family with DRD, underscoring the phenotypic heterogeneity associated with this locus.

**Highlights:**

- A non-coding variant (c.–22C > T) in the 5’ UTR of the *GCH1* gene is identified in a Chinese family with DRD.
- The findings reveal significant clinical heterogeneity within the family, highlighting the complex genotype-phenotype relationship.

## Introduction

Dopa-responsive dystonia (DRD) is a hereditary movement disorder primarily associated with dopamine and/or monamine deficiency [1], with a rare prevalence of approximately 5 per 10 million. Typically, onset occurs between ages 1 and 12, accounting for about 10% of childhood dystonia, though some late-onset cases may present as late as 50–60 years of age [2,3].

The typical DRD phenotype features early-onset dystonia, primarily affecting the limbs and presenting with gait disturbances due to foot flexion and inversion. Dystonia may gradually spread, while cognitive function, cerebellar coordination, sensory processing, and autonomic regulation remain unaffected. Symptoms often exhibit diurnal fluctuations, and low-dose levodopa provides significant symptomatic relief [4,5]. In some cases, DRD may progress to parkinsonism [6,7].

DRD can be inherited in both autosomal dominant and autosomal recessive forms, with the dominant form being more common. The most frequent subtype is caused by autosomal dominant variants in the GTP cyclohydrolase 1 (*GCH1*) gene [8,9,10]. GTP cyclohydrolase 1 is essential for initiating the synthesis of tetrahydrobiopterin (BH4), a key cofactor for three aromatic amino acid hydroxylases, including tyrosine hydroxylase (TH), the rate-limiting enzyme in dopamine synthesis [11]. Variants in *GCH1* can reduce BH4 levels, impaire dopamine synthesis, and disrupt dopaminergic nigrostriatal neurotransmission, leading to, parkinson’s disease, and other related disorders [9,12]. While many coding region variants have been identified, non-coding region variants remain rare [13].

In this study, we described a family pedigree of five individuals diagnosed with DRD and/or parkinsonism and explored the clinical heterogeneity within the family. Whole-exome sequencing (WES) revealed a rare phathogenic variant in the 5’-untranslated region (5’ UTR) of *GCH1* gene. Our findings confirm that non-coding *GCH1* variants can contribute to disease, highlighting the complex genotype-phenotype relationship of *GCH1* variants.

## Methods

### Participants

This study was approved by the Medical Ethics Committee of Xiangya Hospital, Central South University, and was conducted in accordance with the Declaration of Helsinki. All samples were collected from members of a single Chinese family affected by DRD. Informed consent for participation and publication was obtained from the proband and all family members involved in the study.

### Neurological Assessment

Comprehensive neurological examinations were conducted on the proband and selected family members, where feasible, to evaluate both motor and non-motor symptoms. The assessments primarily included the distribution of dystonia, specific tremor types and severity, symptom progression and the presence of other relevant neurological or systemic signs. Key demographic and clinical characteristics were recorded, including age at symptom onset, types and durations of motor symptoms, presence of non-motor symptoms (e.g., hyposmia, neck pain), diurnal fluctuations, and the Babinski sign. Medication responses, particularly to levodopa, and any associated motor complications were also documented. For deceased family members, clinical information was gathered from family reports.

### Whole-Exome Sequencing (WES)

Genomic DNA was extracted from peripheral blood using standard protocols. WES was conducted on the proband (II-10) and five family members (II-7, II-12, III-8, III-9, III-10) to identify potential pathogenic genetic variants. Sequencing libraries were prepared and enriched for exonic regions using an exome capture kit, followed by sequencing on an Illumina platform with an average coverage depth of at least 100x. Sequence reads were aligned to the human reference genome (GRCh37/hg19), and variant calling was performed using a standardized bioinformatics pipeline. Identified variants were annotated and filtered based on population frequency, predicted pathogenicity, and their relevance to neurological disorders. Candidate variants were further validated and assessed for familial co-segregation using Sanger sequencing.

### Sanger Sequencing

Sanger sequencing was performed on the proband and available family members to validate key variants detected by WES. Specific primers were designed to amplify the region of interest in the *GCH1* gene. PCR amplification followed standard protocols, with products purified and sequenced bidirectionally using an ABI Prism 3730 DNA Analyzer (Applied Biosystems). The obtained sequences were aligned to the reference genome (GRCh38/hg38) to confirm the presence of the variant and its co-segregation within the family.

### Database and Bioinformatics Analysis

The identified variants were evaluated for population frequency, clinical significance, and potential functional impact using multiple databases, such as gnomAD and ClinVar, along with predictive tools including CADD, SpliceAI, and PhyloP. Pathogenicity was assessed in accordance with ACMG/AMP guidelines [14], integrating data from population studies, clinical annotations, and functional predictions to ensure a rigorous interpretation.

## Results

### Clinical Characteristics of the Proband

The proband, a 50-year-old Chinese woman, presented with a 23-year history of head and neck deviation with pain, along with over 10 years of limb stiffness and abnormal posture. Two episodes of symptom exacerbation responded well to levodopa treatment. She reported progressive limb stiffness, which significantly impaired her ability to roll over and walk. Physical examination revealed severe abnormal posturing, pronounced muscle rigidity, and tremor (Video 1). Additionally, the patient exhibited marked deficits in fine motor coordination, reduced hand movement speed, and a positive Babinski sign on the left. Autoimmune serology, including anti-nuclear and anti-phospholipid antibodies, was negative. Magnetic resonance imaging findings were unremarkable. Treatment with levodopa (375 mg/day) led to significant symptom improvement within two days. At the one-month and one-year follow-ups, symptoms remained improved, although torticollis persisted (Video 1).

**Video 1:** **Neurological Examination (Initial Visit, 1-Month and 1-Year Follow up): Initial Visit:** The patient is unable to walk and presents with severe resting and postural tremors in both upper limbs, head tremor, and leftward deviation of the head and neck. Fine motor movements in both hands are uncoordinated and significantly slowed. **1-Month and 1-Year Follow up:** The patient is able to walk, with significant improvement in head tremor and upper limb tremors. Fine motor coordination and hand speed have also improved. However, the leftward deviation of the head and neck remains pronounced.

### Clinical Heterogeneity in the Family

**Figure 1:**
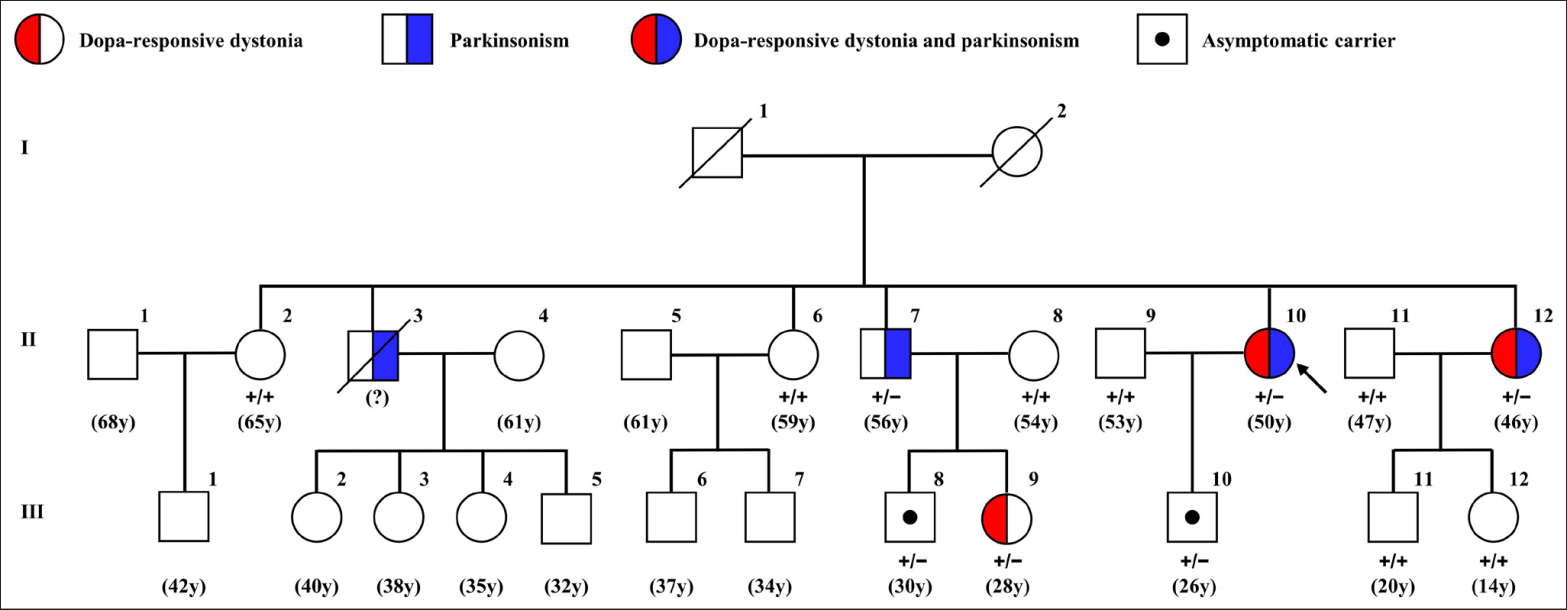
**Pedigree analysis of the *GCH1* gene in the family**. Pedigree illustrating the inheritance pattern, clinical phenotypes, and genotypes of family members. Circles represent females, and squares represent males. Red-filled segments denote individuals diagnosed with dopa-responsive dystonia (DRD), while blue-filled segments indicate individuals diagnosed with parkinsonism. Individuals with both parkinsonism and DRD are shown with a half-blue, half-red symbol. Open symbols indicate unaffected individuals, and dotted symbols represent asymptomatic carriers of the *GCH1* variant. The proband is indicated by an arrow. The question mark (?) beside individual II-3 indicates diagnostic uncertainty due to limited clinical information. Numbers in parentheses denote the age (in years, y) of each individual at the clinical examination. Genotype notation: +/+: Wild-type (no *GCH1* variant detected); +/–: Heterozygous for the *GCH1* variant. Individuals who were not tested for the variant are not labeled with genotypes.

Table 1 highlights the clinical heterogeneity within the family. Patient II-12 exhibited similar clinical features with the proband. Patient II-3 (deceased due to trauma) had bilateral upper limb rest tremor. Patient II-7 experienced both rest and action tremors in the upper limbs for 12 years, and reported hyposmia for 8 years. Patient III-9 had an 18-year history of right foot inversion. Three other affected family members (II-7, II-12, III-9) demonstrated significant symptom improvement with levodopa. No similar symptoms were noted in other family members (Figure 1).

**Table 1:** Demographic and clinical characteristics of affected individuals in the family.


PATIENT	DIAGNOSIS	AGE^a^ (YEAR)	SEX	AGE AT ONSET (YEAR)	MOTOR SYMPTOMS (DURATION, YEAR)	NON-MOTOR SYMPTOMS (DURATION, YEAR)	MUSCLE TONE	DIURNAL FLUCTUATIONS	BABINSKI SIGN	MEDICATIONS	DAILY LEVODOPA DOSE (MG)	DURATION OF LEVODOPA TREATMENT (YEAR)	LEVODOPA RESPONSE	MOTOR COMPLICATIONS OF LEVODOPA

II-3^b^	Parkinsonism ?	N/A	M	N/A	Rest remor of bilateral upper limbs (N/A)	N/A	N/A	N/A	N/A	N/A	N/A	N/A	N/A	N/A

II-7	Parkinsonism	56	M	44	Rest and action tremor of bilateral upper limbs (12)	Hyposmia (8)	Increased	Yes	N/A	Levodopa	N/A	N/A	Yes	No

II-10	DRD Parkinsonism	50	F	27	Cervical dystonia (23); limb rigidity (>10); bradykinesi (>10); rest and action tremor of bilateral upper limbs (>10)	Neck pain (23)	Increased	Yes	Yes	Levodopa; Pramipexole; Eperisone	375	2	Yes	No

II-12	DRD Parkinsonism	46	F	11	Cervical dystonia (35) ; limb rigidity (>20); rest and atcion tremor of bilateral upper limbs (>20)	N/A	Increased	Yes	N/A	Levodopa; Trihexyphenidyl	250	20	Yes	No

III-9	DRD	28	F	10	Right foot inversion (18)	No	Increased	Yes	N/A	Levodopa	62.5	10	Yes	No


^a^: Age at examination. ^b^: According to the family, the patient exhibited rest tremor in bilateral upper limbs but was unable to undergo further examination due to death from trauma. ?: stands for possible. DRD: dopa-responsive dystonia.

### Genetic Findings

WES initially identified a single nucleotide substitution (NM_000161.3: c.–22C > T) in the 5’ untranslated region (5’ UTR) of the *GCH1* gene at genomic position chr14: 54902685 (GRCh38/hg38) in four symptomatic individuals (II-7, II-10, II-12, III-9). The identified variant was further validated by Sanger sequencing, which confirmed its presence in the proband and three additional symptomatic family members. Co-segregation analysis demonstrated that two asymptomatic carriers, III-8 (age 30) and III-10 (age 26), also carried the variant despite showing no related phenotype. The variant was absent in all other unaffected family members.

**Figure 2:**
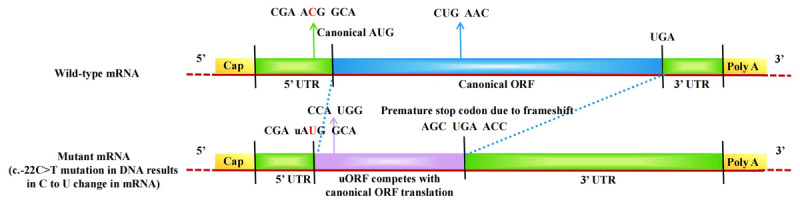
**Comparative schematic of normal individual and mutant GCH1 mRNA**. The upper panel illustrates the normal individual mRNA with translation initiated at the canonical AUG start codon, enabling efficient synthesis of the full-length functional protein. The lower panel depicts the mutant mRNA harboring a c.–22C > T substitution in the 5’-UTR of the *GCH1* gene. This DNA-level substitution (C > T) results in a corresponding RNA change (C > U), creating an upstream AUG (uAUG) codon and initiating an upstream open reading frame (uORF). The presence of the uORF competes with the canonical ORF translation, potentially leading to frameshift variant and a premature stop codon within the coding region. This disruption likely reduces overall protein synthesis, impairs GTP cyclohydrolase 1 production.

This variant introduces an upstream initiation codon (uAUG) and an upstream open reading frame (uORF), which may compete with canonical AUG for translation initiation. Additionally, the variant causes a frameshift mutation, predicted to introduce a premature termination codon (PTC) within the coding region. These mechanisms are likely to reduce BH4 levels and impair dopamine synthesis, potentailly contributing to the disease pathogenesis (Figure 2) [15].

### Variant Classification

Previous studies have demonstrated that the *GCH1* –22C > T variant significantly reduces translation levels [15]. This variant is classified as rare, with a low allele frequency in the gnomAD database (1.4 × 10^–^6). Additionally, the ClinVar database reports this variant (Variation ID: 2925520, Accession: VCV002925520.1) as “pathogenic” or “likely pathogenic.” The incomplete penetrance observed in *GCH1* variants may explain the partial segregation of this variant with disease within the family. Additionally, the PhyloP score of –1.62 indicates that this nucleotide is not highly conserved across species, and the SpliceAI score of 0.00 suggests that this variant is unlikely to affect splicing. Although the CADD score of 4.29 suggests a benign effect, it is important to note that CADD is a predictive tool and may not fully capture all functional consequences observed experimentally or clinically. Based on a comprehensive analysis of functional studies, population genetics, and clinical annotations, and in accordance with ACMG standards and guidelines, we classify the *GCH1* –22C > T variant as “pathogenic” (PS3, PS4, PM2) [14].

## Discussion

The clinical presentation of DRD has been reported to be heterogeneous, ranging from asymptomatic cases to classic dystonia. Initial symptoms may present as arm dystonia, postural tremor, or bradykinesia [16]. Additionally, patients with DRD often experience non-motor symptoms due to reduced tryptophan hydroxylase activity, leading to serotonergic and noradrenergic dysfunction [17,18]. There is evidence suggesting higher rates of depression, anxiety, and obsessive-compulsive disorders in patients with primary dystonia [19,20].

In the studied family, some affected patients exhibited improvement in dystonia in regions other than the neck following oral levodopa therapy, while cervical dystonia remained unresponsive. BH4 is a critical cofactor for the synthesis of neurotransmitters, including dopamine and serotonin. GTP cyclohydrolase deficiency leads to BH4 depletion, which impairs both neurotransmitters [21]. Dysregulation of the central serotonergic system is closely associated with the pathophysiology of cervical dystonia [22]. Therefore, levodopa therapy alone may be insufficient to effectively alleviate cervical dystonia. Based on these findings, we propose that future therapeutic strategies should consider incorporating BH4 supplementation and interventions targeting serotonin synthesis, in combination with dopaminergic agents to more comprehensively mitigate dystonia in affected patients.

DRD is commonly linked to heterozygous variants in the *GCH1* gene. Rare heterozygous *GCH1* variants have been associated with nigrostriatal cell loss, and DRD patients may exhibit additional movement disorders, including parkinsonism and spasticity [17,23,24]. In our study, patient II-7 mainly displayed parkinsonism, while patient III-9 exhibited classic DRD symptoms. Notably, patients II-10 and II-12 initially presented with early-onset DRD but later developed adult-onset parkinsonism, suggesting a broader neurological involvement. These findings highlight the need for long-term follow-up and thorough differential diagnosis.

We identified a –22C > T substitution in the 5’ UTR of the *GCH1* gene. To date, although more than 100 different *GCH1* variants have been reported in DRD, non-coding variants remain rare [10,25]. Previous studies have described three additional single nucleotide substitutions in the 5’ UTR of the *GCH1* gene in DRD patients (–22C > T, –39C > T, and –132T > C), but only the –22C > T variant was showed to be functional, leading to reduced protein expression [15]. The –22C > T substitution affects translational regulation by altering RNA secondary structure, modifying RNA binding protein (RBP) motifs and their interactions, or facilitating the formation of uORFs [26,27,28]. Specifically, this substitution introduces an uAUG and creates an out-of-frame uORF, which may overlap with the physiological ORF (pORF), potentially competing for translation initiation. We hypothesize that the –22C > T substitution in the 5’ UTR of the *GCH1* gene likely reduces GTP cyclohydrolase 1 levels, thereby limiting BH4 biosynthesis and impairing dopamine synthesis, which could contribute to the pathogenesis of DRD and/or parkinsonism. However, these mechanisms remain speculative, and there is currently no direct experimental evidence to support them. Further studies are required to validate these hypotheses and clarify their role in disease pathogenesis [15,29,30].

As noted, individuals III-8 and III-10 in the family were identified as asymptomatic adult carriers of the *GCH1* –22C > T variant, indicating possible incomplete penetrance. This observation aligns with the well-established fact that *GCH1*-associated DRD frequently exhibits incomplete penetrance, particularly in males, whereas females typically demonstrate nearly 100% penetrance [5,17]. The presence of unaffected carriers does not invalidate the pathogenicity of the variant, but rather reflects the complexities of incomplete penetrance. Furthermore, it may suggest that these individuals may not yet have reached the age of onset. Further follow-up studies are warranted to elucidate the clinical significance.

In conclusion, our study identified a rare non-coding variant in the *GCH1* gene in a Chinese family with DRD and/or parkinsonism. The analysis revealed significant phenotypic variability among affected individuals, underscoring the complex genotype-phenotype relationship. Our findings highlight the importance of comprehensive genetic testing, including non-coding regions, in suspected DRD cases lacking typical coding variants. Such an approach enables earlier and more accurate diagnoses, facilitating timely levodopa treatment. While DRD was the primary focus, the implications extend to related disorders like parkinsonism. The role of *GCH1* gene in dopaminergic pathways suggests a potential link to broader neurodegenerative diseases, warranting further investigation into the underlying mechanisms.
